# **Performance improvement of an HVAC system using water and ethylene glycol-based ternary hybrid nanofluids with green-synthesized Fe-Cu-Fe**_**2**_**O**_**3**_ **nanoparticles**

**DOI:** 10.1371/journal.pone.0323539

**Published:** 2025-05-15

**Authors:** Maryam Rabiu Aliyu, Huzaifa Umar, Michael Adedeji, Ali Shefik, Dilber Uzun Ozsahin, Mustafa Dagbasi

**Affiliations:** 1 Energy System Engineering Department, Cyprus International University, Nicosia, Turkey; 2 Operational Research Centre in Healthcare, Near East University, Nicosia, Turkey; 3 Electricals and Electronics Engineering Department, Bahçeşehir Cyprus University, Nicosia, Turkey; 4 Department of Medical Diagnostic Imaging, College of Health Sciences, University of Sharjah, Sharjah, United Arab Emirates; 5 Research Institute for Medical and Health Sciences, University of Sharjah, Sharjah, United Arab Emirates; Government Engineering College Patan, INDIA

## Abstract

This study investigates the potential of water and ethylene glycol-based ternary nanofluids, enhanced with both chemically and green synthesized Fe-Cu-Fe_2_O_3_ nanoparticles, to improve the performance of a heating, ventilation and air conditioning (HVAC) system. It also compares the effectiveness of the two synthesis methods, addressing the current research gap in the practical application of green-synthesized nanofluids in HVAC systems. Two sets of nanoparticles were synthesized using chemical and eco-friendly methods and dispersed in a base fluid of water and ethylene glycol (EG) at varying concentrations of 0%, 50%, and 75% EG with various nanoparticle mixture ratios. The prepared ternary nanofluids were used to evaluate the thermal performance of the heater in an air-handling unit (AHU). Experimental data on the thermophysical properties of the nanofluid at the different mixture ratios were incorporated into a numerical model simulating the AHU operating in a Mediterranean climate. Results show that the nanofluids significantly enhance system performance, with the 1Fe: 2Cu: 1Fe_2_O_3_ nanoparticle mixture ratio offering the best balance between efficiency and operational stability. The heat transfer rates were improved by up to 20% in summer and 15% in winter compared to the base fluid. In addition, the mixture ratio achieved optimal exergy efficiency, peaking at 98% during winter operation and 96% during summer. Comparative analysis also shows that the ternary nanofluids containing the chemical-synthesized nanoparticles (CSNTNF) perform only slightly better than the nanofluids containing the green-synthesized nanoparticles (GSNTNF). This suggests that GSNTNF is a suitable replacement for the CSNTNF, considering the environmental benefits.

## 1. Introduction

In today’s world, air conditioning (AC) systems are becoming increasingly essential, given that they enable people to live or work in suitable environments. The main contributors are rising temperatures brought on by global warming and improved living standards [1].

Heating, ventilation, and air conditioning (HVAC) systems, however, are among the highest energy consumers in residential, commercial, and industrial applications, accounting for nearly 50% of total building energy use [2]. Improving HVAC systems’ thermal performance is essential for reducing energy consumption and mitigating GHG (greenhouse gas). HVAC systems’ very high energy consumption has necessitated identifying innovative methods to lower building energy use without compromising indoor air quality or well-being [3]. Multiple techniques that HVAC systems may apply to increase their energy efficiency and lower their ecological impact have been explored. A standard method researchers have extensively studied is utilizing control and optimization approaches to lower these systems’ energy usage rates. These strategies range from intelligent control systems and machine learning applications to predictive control methods and optimization algorithms [4]. Various studies have been conducted on such strategies as real-time data acquisition and machine-learning algorithms [5]. Model Predictive Control (MPC) algorithms [6] and Advanced optimization algorithms, such as the Ant Lion Optimizer with Enhancements (ALOE) and Double Dueling Deep Q-learning (D3QN), have been used for this purpose [7].

Another method of improving HVAC systems’ performance is through efficiency advancements in heat transfer processes. This can be achieved by utilising novel heating/cooling fluids to lower the heating system’s total size and energy consumption while improving its thermal performance. Nanotechnology has attracted much attention lately for its potential to improve the HVAC industry’s heat transfer, coolant efficiency, and primary and secondary refrigerant efficiency. Nanofluids (NFs) are specially designed heat transfer fluids that may be applied to multiple chillers, heat pumps, and other hydronic HVAC systems to improve heat transfer and thereby increase energy efficiency. NFs can improve the performance of HVAC sector heat recovery systems and air and liquid heat exchangers comprising condensers and evaporators. Nanofluids represent a novel kind of heat transfer fluid with high thermal performance for various applications. The size of nanoparticles is under 100 nm and they are distributed in a base fluid, like ethylene glycol, water, or propylene glycol. The thermal conductivity of such mixes is increased by adding high thermal-conductivity metallic nanoparticles (such as copper, aluminium, silicon, and silver), improving their total capacity to transfer heat energy [8]. NFs are widely utilized across various applications due to their distinctive properties, including improved thermal conductivity, convective heat transfer, and mass transfer [9]. They are commonly employed in heat exchangers, cooling systems, and electronic devices to enhance thermal performance [10]. Maxwell was the first to perform research to increase the ability of liquids to transfer heat through solids particles in 1873, which signified the start of the history of these heat-transfer fluids. Over a century before, Choi and Eastman (1995) proved that the addition of metallic nanoparticles (NPs) into the base fluid substantially increases its thermal conductivity. Subsequent studies have explored the role of nanoparticle size, shape, material, and concentration on the thermophysical properties of NFs [11].

NFs are advanced heat transfer fluids created by dispersing NPs, typically falling between 0 and 100 nanometers in size, into conventional base fluids such as ethylene glycol (EG), water (W), and ethylene glycol-water mixtures (EG-W) and oils [12]. These include oxides, nitrides, carbides, metals, carbon-based materials, or hybrid NPs [13]. NFs containing 0.01g/L of TiO_2_ with a particle size of 20nm prepared using the thermal oscillation method to influence the breakdown voltage of transformer oil under severe cold conditions [14]. Recent studies on the thermophysical properties of NFs highlight that multiple factors influence their thermal conductivity and dynamic viscosity. Key parameters include the Brownian motion of NPs, particle size and shape, the type of NPs and base fluid used, nanofluid temperature (T), particle volume fraction (ϕ), and the presence of surfactants. These characteristics collectively determine NFs’ performance and application potential in various heat transfer and energy systems [15]. Integrating nano-Fe₂O₃-based nanofluid significantly enhanced thermal and electrical performance in PV/T systems. Both monocrystalline and polycrystalline PV panels showed high efficiency and exergy output, making them viable solutions for use in harsh weather conditions [16].

Researchers have reported substantial improvements in heat transfer efficiency using NFs in thermal systems. For instance, a study using computational fluid dynamics (CFD) simulations demonstrated that the addition of NFs to a base fluid increased the heat transfer coefficient, thereby enhancing heat transfer performance in a 2D tube setup [17]. In electronic cooling systems, NFs’ thermal conductivity plays a vital role in heat dissipation, preventing overheating and ensuring efficient operation. Chaichan et al. reported that MWCNT-based nanofluids significantly improved the thermal and electrical efficiency of PV/T systems, especially in harsh summer conditions, and a nanofluid-cooled PV/T system outperformed both standalone PV and water-cooled PV/T, making it a viable solution for extreme climates like Baghdad [18]. Arif et al. showed that using water-based ternary hybrid nanofluid enhances the heat transfer rate by up to 33.67% compared to water as a heat transfer fluid in an automobile radiator [19].

Several studies have also investigated the potential of NFs to improve the thermal performance of HVAC systems. Ahmed and Khan found that a nanofluid containing Al_2_O_3_ with a volume fraction of 5% can enhance the coefficient of performance (COP) of an air conditioner by as much as 22.1%. However, copper nanofluid revealed a more remarkable improvement of 29.4% [20]. A study by Milanese et al. on HVAC systems also showed that the COP of a heat pump with nanofluid improved by 9.8% on average during the winter and 8.9% during the summer [3]. Similarly, Colangelo et al. reported a 10% increase in the HVAC system’s performance when utilizing water-glycol nanofluid with NPs of Al_2_O_3_ [21]. Various factors enhance thermal conductivity in hybrid NFs, with the mixture ratio being a key parameter. The mixture ratio, which defines the proportion of each type of NP in the completed solution, significantly influences the interaction and coordination between the 2,3 nanoparticle types. This latter interaction reduces the spacing between larger particles, ultimately impacting critical thermal properties such as thermal conductivity [22]. Sundar et al. investigated the heat transfer properties of MWCNT-Fe₃O₄ hybrid NFs within a range of 0.1% to 0.3% of particle loading [23]. Their study found that the optimal thermal conductivity, measuring 0.7656 W/mK, was achieved at 0.3% concentration in volume and at a temperature of 40 °C, reflecting a thermal conductivity increase by 27.18% [24].

Ternary NFs, which combine multiple types of NPs, can potentially offer synergistic effects, further enhancing thermal and rheological properties. For instance, a study on Al₂O₃-TiO₂-SiO₂ ternary nanofluids demonstrated a thermal conductivity enhancement of 24.8% at a 3.0% volume concentration, which is higher than typical enhancements seen in binary nanofluids [25]. In another study, the ternary hybrid NFs composed of Cu-CuO-Al₂O₃ in water showed superior heat transfer efficiency, outperforming both NFs and hybrid NFs models in a quadrantal enclosure setup [26]. The addition of zirconium oxide to a graphene oxide and cobalt binary NFs resulted in ternary NFs that increased thermal conductivity by up to 20% with a 0.1 volume fraction, demonstrating a significant enhancement in thermal performance [27]. Furthermore, ternary NFs display improved dispersion stability, decreasing the likelihood of sedimentation over prolonged periods. The stability of these fluids is often superior to that of binary NFs, as demonstrated in studies where ternary NFs maintained better suspension stability and lower viscosity, which are desirable for fluid flow characteristics [28]. These properties make them ideal for high-performance heat exchangers in HVAC systems.

Hybridization has significantly improved heat transfer properties and valuable applications of NFs. Numerous reviews have focused on the enhanced thermophysical properties of hybrid NFs, highlighting their potential in various applications [29]. Cakmak et al. investigated the properties of ternary nanocomposites made with sol-gel of rGO-Fe₃O₄–TiO₂ dispersed in ethylene glycol (EG) [30]. They employed Zeta potential, SEM, EDX, FTIR, and XRD analyses to examine the morphology and stability of the nanocomposites. Thermal conductivity measurements were conducted over a 25–60°C temperature range, revealing a strong correlation between temperature-related variables and volume fraction increases in thermal conductivity. The study demonstrated a 13.3% increase at 60°C thermal conductivity with a 0.25 wt% NFs concentration. The ternary hybrid NFs also showed sufficient stability, making them suitable for heating and cooling applications [31].

Despite the promising potential of NFs, challenges remain. Many studies rely on chemically synthesized NPs, often involving toxic reagents and environmentally harmful processes. Green synthesis methods, which use plant-based reducing agents or other eco-friendly techniques, have emerged as a sustainable alternative. These methods minimise environmental impact and produce NPs with improved biocompatibility and stability. The synthesis of NPs traditionally involves chemical methods that pose environmental and health risks due to using hazardous chemicals. Green synthesis methods have gained traction as a sustainable alternative in recent years. These methods utilize plant extracts, bacteria, or fungi as reducing agents to synthesize NPs in an environmentally benign manner. Studies have demonstrated that green-synthesized NPs exhibit comparable or superior thermal and structural properties to chemically synthesized ones. For example, green NFs incorporating these NPs have significantly increased thermal conductivity. One study reported an improvement of 11.3% in thermal conductivity compared to distilled water without a significant increase in fluid viscosity [32]. Another study found that green NFs with a concentration of 0.3% exhibited a maximum thermal conductivity increase of 17% over the base liquid [33]. However, applying green-synthesized NPs in ternary NFs remains underexplored, creating a research gap this study aims to address. However, applying green-synthesized ternary NFs in practical systems, such as HVAC, is still underexplored.

In addition, the performance of HVAC systems is significantly influenced by climatic conditions. The Mediterranean region, characterized by hot summers and mild winters, presents unique challenges for maintaining energy efficiency and indoor comfort. While the influence of climatic conditions on HVAC system performance is widely acknowledged, there is limited research evaluating the role of advanced heat transfer fluids, such as ternary NFs, in addressing the unique challenges of the Mediterranean region’s climate. In most of the reviewed literature on ternary NFs, researchers primarily focus on examining the effects of nanoparticle volume concentration and temperature on the thermal and physical properties of ternary NFs. However, it is essential to recognize that the nanoparticle mixing ratio plays a critical role in developing ternary NFs with excellent dispersion stability and enhanced thermophysical properties.

To address these gaps, this study investigates the performance enhancement of an HVAC system utilizing water and ethylene glycol-based ternary NFs prepared at various mixing ratios (0%, 50%, and 75% concentrations) and evaluates their thermal conductivity, dynamic viscosity, dispersion and pH stability. It compares the performance of chemically synthesized NPs and green-synthesized NPs and their effectiveness in the synthesis of ternary NFs. The synthesized NPs were incorporated into an ethylene glycol-water mixture at different base fluid concentrations and nanofluid volume fractions. The experimental analysis focuses on evaluating the thermal performance of the heater in an air-handling unit (AHU). The experimental analysis focuses on assessing the thermal performance of a heater in an air-handling unit (AHU), with results integrated into a numerical model simulating AHU operation under typical Mediterranean climate conditions. The numerical analysis was used to evaluate the impact of the synthesized NFs on the performance of an HVAC system and determine the optimal mixture ratio for maximum performance. A comparative analysis was carried out to determine the performance differences between chemically synthesized ternary nanofluids (CSNTNF) and green-synthesized ternary nanofluids (GSNTNF).

## 2. Experimental methodology

### 2.1 Synthesis of the metal and the metallic oxide nanoparticles

**Fig 1:**
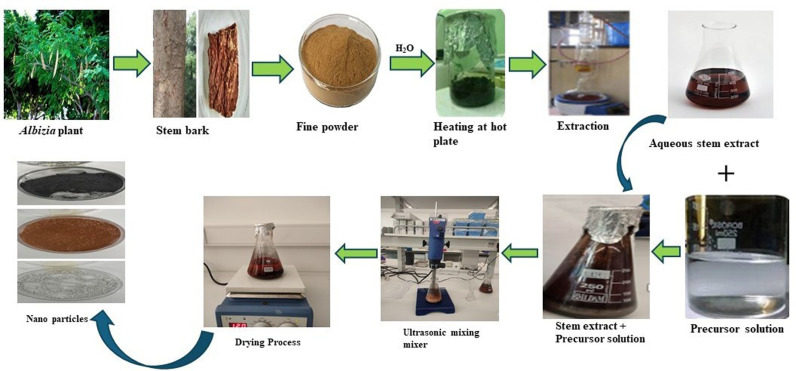
Illustration of the experimental process involved in the synthesis of the metallic and metallic oxide nanoparticles prior to the preparation of the nanofluids.

Chemical and green approaches have been used to synthesize Iron Nanoparticles (Fe NPs), Copper Nanoparticles (Cu NPs), and Iron Oxide Nanoparticles (Fe_2_O_3_ NPs) using the microemulsion technique at controlled temperatures and pH levels. Fig 1, illustrates the summary of the synthesis process.

The synthesis of Fe NPs was conducted using a Fe² ⁺ solution, with green synthesis employing the same solution and *Albizia lebbeck* extract in a 10:2 volume ratio, following the method of Petcharoen and Sirivat (2012) with slight modifications. The stem bark of *Albizia lebbeck* was dried, ground into a coarse powder, and extracted as described by Huzaifa et al. [34]. For the green synthesis, 0.5 M FeSO₄·7H₂O solution was mixed and continuously stirred for 30 minutes at 60°C. The resulting Fe NPs were collected, rinsed with deionized water, and dried in a vacuum oven at 80°C for 10 hours. A colour change from yellow to black was observed immediately after the plant extract was added, indicating the successful formation of Fe NPs.

The synthesis of Cu NPs was performed using a copper (II) sulfate pentahydrate (CuSO₄·5H₂O) solution. The green synthesis involved utilizing the same solution with a plant extract as a stabilizing agent in a 10:2 volume ratio, as described by Din et al. [35]. The procedure began by adding 0.5 M copper (II) sulfate pentahydrate solution to the plant extract under vigorous stirring and reflux for 50 minutes. This was followed by the addition of 0.2 M ascorbic acid solution. Subsequently, 30 mL of 1 M sodium hydroxide solution was gradually introduced into the mixture while maintaining constant stirring and temperature at 80 °C for 5 hours. A colour change from greenish to yellow confirmed the formation of Cu NPs. The particles were cleaned and dried at room temperature.

The Fe_2_O_3_ NPs synthesis was carried out with 0.5M ferric chloride (FeCl_3_) solution, and the green synthesized Fe_2_O_3_ NPs using the same solution and the plant extract in 10:2 volume ratio respectively, using the method of Huzaifa et al. [36]. After being centrifuged at 10,000 rpm for 30 minutes to achieve complex formation, the mixture underwent 2–3 washes with Millipore water to eliminate impurities and kept at 120 °C for 10 consecutive hours.

### 2.2 Nanofluids preparation

**Fig 2:**
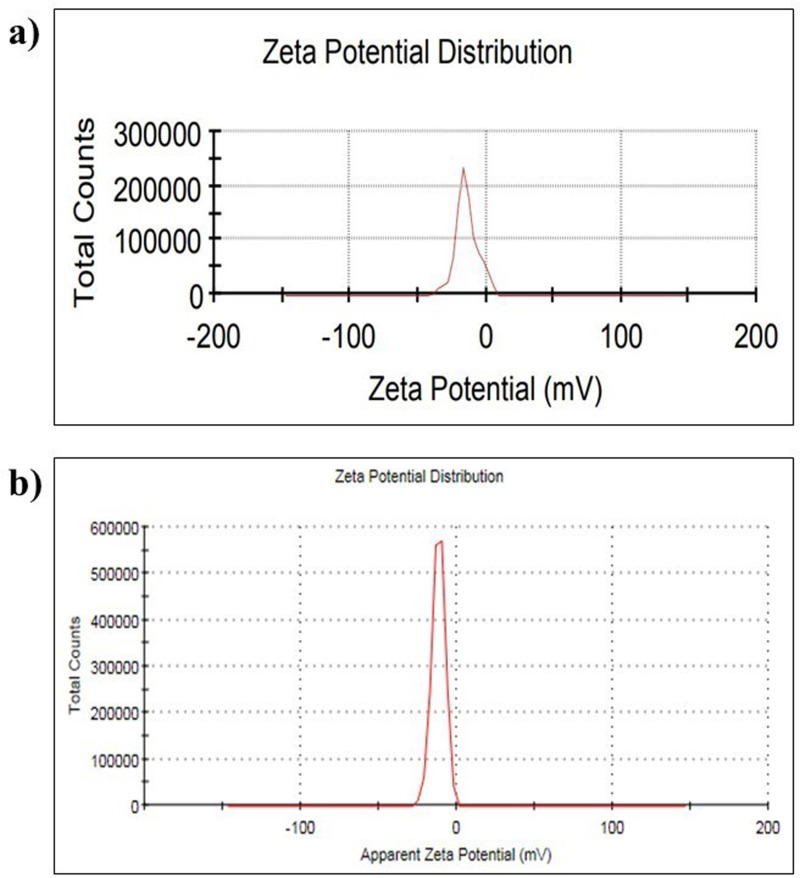
Zeta potential distribution of the prepared a) CSNTNF, and b) GSNTNF.

The ternary NFs were prepared with two different base fluids using the two-step procedure reported by Hawwash et al. with slight modification [37]. The NPs were dispersed in a 100:0, 50:50, and 75:25 water-ethylene glycol mixture at volume concentrations of 0%, 0.5%, and 0.75%, respectively. An ultrasonic bath Sonicator, magnetic stirrer and pH meter were used to obtain stable NFs. Ultrasonication for 45 mins was carried out to ensure homogeneity. Stability tests, including zeta potential measurements, were conducted using a Malvern Zetasizer Analyser (ZSU310) equipped with Zetasizer Ultra Pro ZS Xplorer software to confirm dispersion quality, and the result revealed an improved zeta potential of the synthesized NFs (Fig 2a-2b). The Experimental results (Table 1) indicate that GSNTNF (green-synthesized ternary nanofluids) generally exhibit more excellent stability than CSNTNF (chemically synthesized ternary nanofluids), as evidenced by their higher zeta potential values. Among all tested compositions, nanofluids prepared with 75% ethylene glycol demonstrated the highest zeta potential, indicating enhanced colloidal stability. Notably, the zeta potential reached a peak value of 55 mV for the 2Fe:3Cu:1Fe₂O₃ mixture ratio synthesized using *Albizia lebbeck* extract, highlighting the effectiveness of the green synthesis method in producing stable nanoparticles (Fig 2a). Additionally, the average particle sizes of the Fe, Cu, and Fe₂O₃ nanoparticles used in the nanofluid formulations were 21 nm, 36 nm, and 24.7 nm, respectively.

**Table 1:** Zeta Potential of the CSNTNF and GSNTNF for different mixture ratios of Fe:Cu:Fe_2_O_3_, and base fluid compositions.

Ethylene Glycol	1:1:1 (mV)	2:1:1 (mV)	1:2:1 (mV)	1:1:2 (mV)	3:2:1 (mV)	1:2:3 (mV)	2:3:1 (mV)
0% CSNTNF	40	43	42	44	45	47	49
0% GSNTNF	42	44	44	45	46	48	49
50% CSNTNF	40	43	41	44	47	49	50
50% GSNTNF	40	43	41	44	47	49	50
75% CSNTNF	45	47	46	48	50	50	52
75% GSNTNF	45	47	46	48	50	50	55

### 2.3 Characterization of the nanofluids

The synthesized NPs were washed, dried, and characterized using various spectroscopic and microscopic techniques.

**Fig 3:**
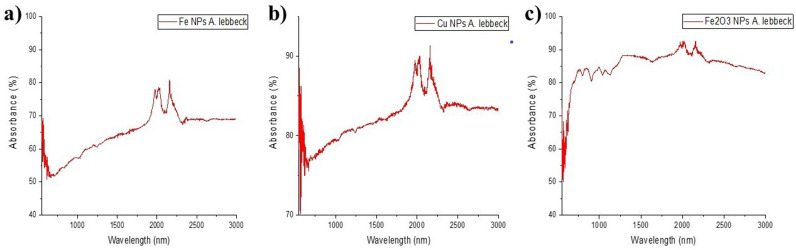
FTIR spectra of (a) Fe NPs, (b) Cu NPs, (c) Fe_2_O_3_ NPs .

Identification of the functional groups in the NPs by employing a Fourier Transform Infrared Spectrophotometer (FTIR) operated at a frequency range of 500–3000 cm^−1^ revealed organic compounds such as primary saturated alcohol (C–O), aliphatic carboxylic acid (C = O), methyl group (C = CH), and water molecules (O-H), that can enhance the activity of the NPs by preventing agglomeration and stabilizing the NPs (Fig 3).

**Fig 4:**
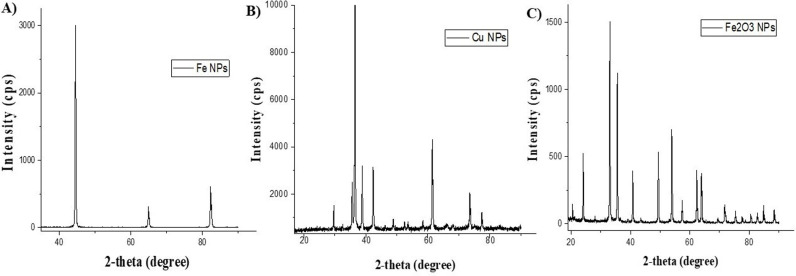
XRD pattern (A) Fe NPs, (B) Cu NPs, (C) Fe_2_O_3_ NPs.

The Rigaku ZSX Primus II X-ray diffractometer (XRD) determined each NP’s crystalline nature. This required analyzing the NPs’ structural properties using powdered samples in an X-ray diffractometer outfitted with CuK radiation. Fig 4 shows the XRD spectra of the green synthesized Fe, Cu, and Fe_2_O_3_ NPs. The peaks revealed by the synthesized NPs are within the range of the peaks exhibited by metal particles, and they confirmed the formation of purified Fe, Cu, and Fe_2_O_3_ NPs, respectively.

**Fig 5:**
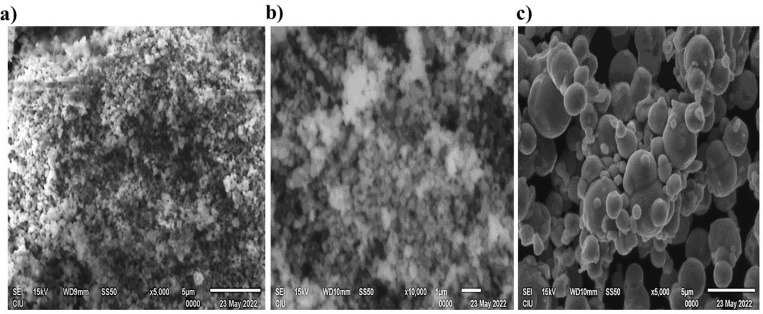
SEM images of the synthesized nanoparticles using *Albizia lebbeck* extract: (a) Fe NPs, (b) Cu NPs, (c) Fe_2_O_3_ NPs.

**Fig 6:**
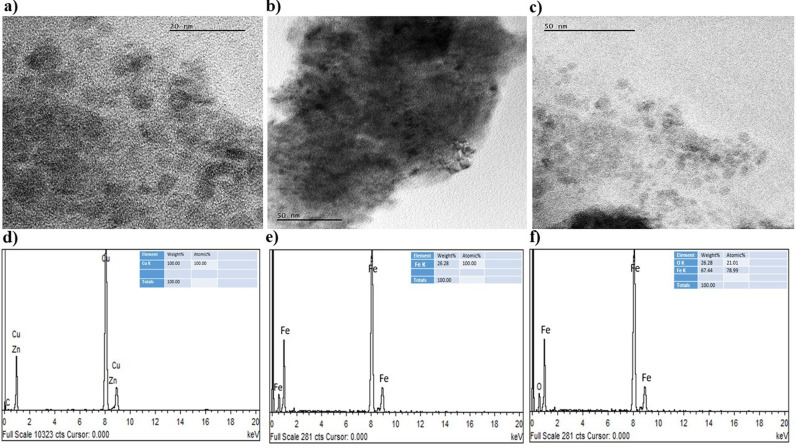
TEM images of: (a) Fe NPs, (b) Cu NPs, (c) Fe_2_O_3_ NPs. EDS spectra of: (d) Fe NPs, (e) Cu NPs, (f) Fe_2_O_3_ NPs.

Furthermore, the morphological structure of the NPs was investigated using a scanning electron microscope (SEM) (JOEL JSM 6335-F) and transmission electron microscope (TEM) using High-Resolution Transmission Electron Microscopy (JEOL JEM 2100 HRTEM) at 200 kV. The surface appeared to be irregularly spherical, and Fe, Cu and O were detected from the elemental mapping conducted using energy-dispersive X-ray spectrometry (EDS) equipped with Gatan Model 833 Orius SC200D and Gatan Model 794 Multiscan CCD Camera as shown in Figs 5(a-b) and 6(a-f).

## 3. HVAC system model

### 3.1 System description

**Fig 7:**
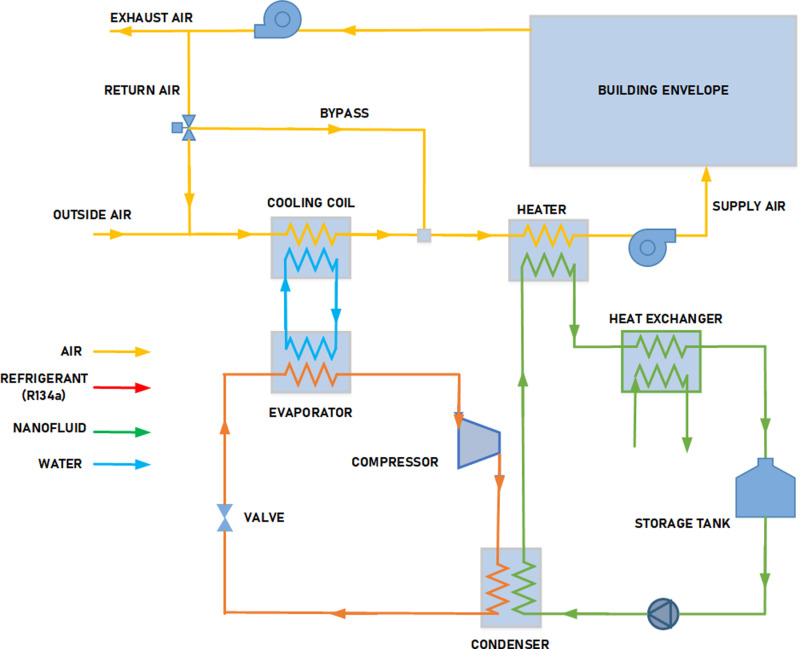
Schematic diagram of the HVAC system.

The key components of the modelled system are air-conditioned rooms and air handling units (AHU); the latter include a cooling and dehumidifying coil, filter, heating coil, fan, humidifier, and ducting, as shown in Fig 7. The winter and summer operating seasons are also considered.

Hot, moist air enters the AHU’s dehumidification and cooling coil during the summer operating season. As air moves through a coil, the temperature falls, and water moisture condenses, lowering the moisture content of the air. Once the zone’s temperature is detected, the thermostat notifies the controller to take the necessary steps and instructs the chiller to raise or lower the water temperature fed to the cooling foil. For dehumidification, the cold energy is supplied to the cooling coil by a vapour compression chiller. The humidity ratio in the area is monitored by a humidistat, which instructs the chiller to lower the water temperature in terms of high relative humidity values so that the water vapour in the humid air condenses and the humidity ratio of the supply air is reduced. The cold, dehumidified air then passes through the heater to increase its temperature to the desired supply temperature value. The heat energy supplied to the heater is extracted from the condenser of the vapour compression chiller. The TNF considered in the present study operates as both the cooling fluid of the vapour compression chiller condenser and as the heating fluid of the heater. The TNF flowing through the condenser extracts heat from the hot refrigerant in the vapour compression chiller. The TNF then flows through the heater to supply the heat needed to raise the temperature of the dehumidified supply air to the required level.

The AHU receives cold, dry air throughout the winter operating season. The TNF is again utilized to heat the incoming cold supply air. Lastly, the air flows through the humidifier, which produces vapour to regulate humidity. Dampers and/or variable-flow fans regulate the system’s airflow rate.

### 3.2 Mathematical modelling

Thermal energy is added to or removed from the conditioned airflow throughout the heating and cooling process to achieve optimum moist air temperature levels. This energy used for conditioning is evaluated per Equation 1 during the occurrence of the process of heating and Equation 2 when the air conditioner cools the moist air:


$${\dot{Q}}_{heat}={\dot{m}}_{air}\left(h_{out}-h_{in}\right)$$ (1)

$${\dot{Q}}_{cool}={\dot{m}}_{air}\left(h_{in}-h_{out}\right)$$ (2)

where ${\dot{m}}_{air}$ is the air mass flow rate that needs to be conditioned and $h_{in}$ and $h_{out}$ are the initial and final specific enthalpy of the humid air, with the initial and final with respect to cooling/heating units.

When the heating/cooling operation is completed, the temperature of the moist air is brought to a higher/lower value. On the other hand, a humidification/dehumidification procedure will be carried out to attain the required humidity ratio of the supply air. Thus, regarding the air’s water content, the conditioning plant transfers energy to and from the mowing air based on the water content, which provides/removes the energy to/from the mowing air. The energy input rates are computed from Equation 3 and Equation 4, where the humidification/ dehumidification unit is used to refer to the initial and final enthalpy, and the necessary dehumidification energy considers both latent energy and sensible contributions:


$${\dot{Q}}_{dehumidification}={\dot{m}}_{air}\left(h_{out}-h_{in}\right)$$ (3)

$${\dot{Q}}_{humidification}={\dot{m}}_{air}\left(h_{in}-h_{out}\right)$$ (4)

In the hot season, after completing the dehumidification process, the air is close to the dew point, and reheating is generally applied to bring the air temperature to the desired supply temperature. The reheat energy is calculated using equation 5:


$${\dot{Q}}_{reheat}={\dot{m}}_{air}\left(h_{out}-h_{in}\right)$$ (5)

The system’s overall heat exchange coefficient varies when various heat transfer fluids are used because thermo-physical properties vary depending on the convective heat transfer coefficient and heat transfer fluid’s chemical and physical composition variation. The following equation 6, which is analogous to Ohm’s law, links the system’s global heat transfer coefficient (U) to its total thermal resistance $R_{t}$.


$$R_{t}=\frac{1}{UA}$$ (6)

The convective heat transfer coefficient (h) will depend on the type used of heat transfer fluids following equation 7, it depends upon the fluid’s thermal conductivity ($k_{f}$), Renold number (Re) and Nusselt number (Nu) [38]:


$$h=\frac{Nuk_{tnf}}{D}$$ (7)

$$Re=\frac{\rho_{f}vD}{\mu_{tnf}}$$ (8)

$$Nu=0.021{Re}_{tnf}^{0.8}{\mathrm{Pr}}_{tnf}^{0.3}$$ (9)

Subsequently, using equations, the model evaluates whether the hot or the cold side has the lowest capacitance (Equations 10 and 11), after which effectiveness is computed using the given flow setup and on UA by Equation 13 [39]:


$$C_{cooling}={\dot{m}}_{c}C_{p,c}$$ (10)

$$C_{heating}={\dot{m}}_{h}C_{p,h}$$ (11)

$$\varepsilon =1-exp\left[{\left(\frac{C_{min}}{C_{max}}\mathrm{\backslash rightleft}(\frac{UA}{C_{min}}\right)}^{0.22}\left\{\exp\left[\left(\frac{{-C}_{min}}{C_{max}}\right){\left(\frac{UA}{C_{min}}\right)}^{0.78}\right]-1\right\}\right]$$ (12)

The pressure drop of the TNF (${\Delta p}_{f}$) as it moves through the heater, can be calculated using equation 13 [40]:


$${\Delta p}_{tnf}=\frac{\left[G_{tnf}^{2}\times f_{tnf}\times H_{c}\right]}{\left[2\times \rho_{tnf}\times \left(\frac{D_{h,tnf}}{4}\right)\right]}$$ (13)

Where friction factor ($f_{tnf}$) is evaluated by existing correlations by Equation 13 [41].

The pumping power (*P*_*P*_) for the coolant side is calculated by Equation 14 (Sahoo, 2020b):


$$P_{P}={\dot{V}}_{f}\times {\Delta p}_{tnf}$$ (14)

The thermal performance factor (TPF) of the system is defined as Equation 15 (Sahoo, 2017):


$$TPF=\frac{Nu}{N_{s}}$$ (15)

The $N_{s}$ can be determined using Equation 16 [42]:


$$N_{s}=\frac{S}{gen}C_{\max}$$ (16)

The entropy generation (${\dot{S}}_{gen}$) is determined by Equation 17 (Sahoo, 2017):


$${\dot{S}}_{gen}={\dot{m}}_{a}\left[c_{p,a}\ln\frac{T_{a,e}}{T_{a,i}}-R_{a}\ln\frac{P_{a,e}}{P_{a,i}}\right]+{\dot{m}}_{f}\left[c_{p,tnf}\ln\frac{T_{tnf,e}}{T_{tnf,i}}-\frac{P_{a,e}-P_{a,i}}{\rho_{tnf}T_{tnf,mean}}\right]$$ (17)

The TNF exergy loss (${\Delta Ex}_{f}$) is calculated by Equation 18 (Sahoo & Sarkar, 2017):


$${\Delta Ex}_{tnf}=\dot{Q}-T_{0}\left[{\dot{m}}_{tnf}C_{p,tnf}\ln\left(\frac{T_{tnf,in}}{T_{tnf,out}}\right)-\frac{{\dot{m}}_{tnf}{\Delta p}_{tnf}}{\rho_{tnf}T_{tnf,mean}}\right]$$ (18)

By contrast, the exergy rate gained by air (${\Delta Ex}_{air}$is calculated by Equation 19 (Sahoo et al., 2017):


$${\Delta Ex}_{air}=\dot{Q}-T_{0}\left[{\dot{m}}_{air}C_{p,air}\ln\left(\frac{T_{air,in}}{T_{air,out}}\right)+{\dot{m}}_{air}R\ln\left(\frac{p_{air,in}}{p_{air,out}}\right)\right]$$ (19)

Exergetic efficiency ($\eta_{II}$) is expressed as Equation 20 [39]:


$$\eta_{II}=\frac{{\Delta Ex}_{air}}{{\Delta Ex}_{tnf}}$$ (20)

An engineering equation solver (EES) code has been developed for the previously discussed coupled equations to evaluate the theoretical results.

### 3.3 Ambient conditions and design parameters

The operation of the HVAC system’s Air Handling Unit (AHU) will be evaluated under both winter and summer conditions to assess seasonal performance variations. Specific design parameters and ambient conditions reflective of each season will be considered to simulate real-world operational scenarios. For this study, the climatic conditions of Cyprus International University, located in the Turkish Republic of Northern Cyprus (TRNC) at coordinates 35.27°N latitude and 33.06°E longitude, were selected as the reference site.

**Fig 8:**
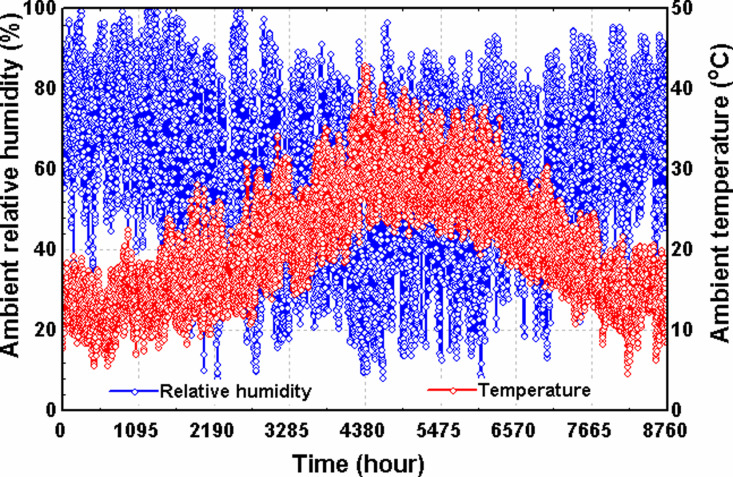
Ambient conditions for the selected location (longitude33.06°E, latitude 35.27°N).

As illustrated in Fig 8, the ambient temperature at this location can fall below 10°C during the winter months, accompanied by a high relative humidity (RH) that often exceeds 90%. In contrast, temperatures can soar up to 40°C during the summer, while RH can drop to less than 20%. These environmental patterns are characteristic of a Mediterranean climate, which presents distinct HVAC challenges across seasons. Specifically, the high temperatures and low humidity in summer necessitate efficient cooling and dehumidification to maintain indoor thermal comfort. At the same time, the colder, more humid winter conditions require reliable heating and humidity control. Therefore, the system must be optimized to perform effectively under both extremes to ensure year-round indoor environmental quality and energy efficiency.

Table 2 displays the proposed input conditions for the steady state analysis of the system for winter and summer. The selected winter ambient temperature and RH are 10^o^C and 70%, respectively. For the summer period, the ambient temperature and RH are taken as 35^o^C and 30%, respectively. The indoor supply air is set as around 28^o^C during winter and 17^o^C during summer. Although the optimum indoor comfort temperature is known to be between 22^o^C and 26^o^C, these design values in Table 2 have been chosen to ensure that the system will be able to provide sufficient heating and cooling if needed. The supply air RH is also set as 55% for both winter and summer seasons. The temperature of the return air drawn out of the building is set as 23^o^C. The bypass ratio, which will determine the fraction of the return air that will be recirculated into the AHU is set as 0.3 for both winter and summer seasons.

**Table 2:** Design conditions for the system operation.

Design condition	Winter	Summer
Ambient air temperature (^o^C)	10	35
Ambient air relative humidity (%)	70	30
Ambient air humidity ratio (g/g)	0.0053	0.0105
Supply air temperature (^o^C)	28	17
Supply air volume flow rate (m^3^/s)	2	2
Supply air relative humidity (%)	55	55
Return air temperature (^o^C)	23	23
Bypass ratio	0.3	0.3

## 4. Results and discussion

This section presents and analyzes the results from the experimental and numerical evaluations of the HVAC system performance enhanced with ternary NFs. Key findings on the thermophysical properties of the NFs, their impact on air-handling unit (AHU) performance, and a comparison of chemically synthesized (CSNTNF) and green-synthesized (GSNTNF) NFs are discussed.

### 4.1 Thermophysical properties of the CSNTNF

The thermophysical properties of the synthesized NFs (CSNTNF and GSNTNF) were determined, and the results were recorded. Specific heat capacity, thermal conductivity, density, and viscosity were the thermophysical properties considered in our studies.

The specific heat capacity of the prepared NFs was measured using a Differential Scanning Calorimeter (METTLER TOLEDO, DSC-60) with a heating rate of 10ºC/min, a temperature range of 25–100°C, and an isothermal duration of 10 min. Specific heat capacity is a key physical property significantly impacting heat transfer processes. Extensive research has been conducted on the specific heat capacity of CSNTNF and GSNTNF to evaluate their impact on enhancing heat transfer efficiency (Table 3).

**Table 3:** Specific heat capacity of the CSNTNF and GSNTNF for different mixture ratios of Fe:Cu:Fe_2_O_3_ and base fluid compositions (Ethylene Glycol) at 25^o^C.

Ethylene Glycol	1:1:1 (J/g K)	2:1:1 (J/g K)	1:2:1 (J/g K)	1:1:2 (J/g K)	3:2:1 (J/g K)	1:2:3 (J/g K)	2:3:1 (J/g K)
0% CSNTNF	3280	3705	3285	3294	3423	3253	3536
0% GSNTNF	3291	3750	3300	3320	3451	3268	3291
50% CSNTNF	3420	3750	3342	3315	3478	3293	3596
50% GSNTNF	3291	3750	3300	3320	3451	3268	3543
75% CSNTNF	3510	3795	3398	3368	3506	3385	3640
75% GSNTNF	3291	3750	3300	3320	3451	3268	3543

The thermal conductivity of the NFs was determined using the Thermal Conductivity Tester (DRE-2B). The probe sensor was inserted into various mixture ratios of CSNTNF and GSNTNF, with results presented in Table 4. Measurements were conducted across a temperature range of 20 °C to 50 °C by initially heating the nanofluid samples from room temperature to 60 °C, followed by gradual cooling under ambient air conditions. The instrument operates by supplying a known current to the sensor’s heating component, generating small quantities of heat. This heat causes a temperature increase at the interface between the sample and the sensor, leading to a voltage drop in the sensor. This voltage drop is used to calculate the thermal conductivity of the tested samples.

**Table 4:** Thermal conductivity of the CSNTNF and GSNTNF for different mixture ratios of Fe:Cu:Fe_2_O_3_, and base fluid compositions at 25^o^C.

Ethylene Glycol Concentration	1:1:1 (W/m.k)	2:1:1 (W/m.k)	1:2:1 (W/m.k)	1:1:2 (W/m.k)	3:2:1 (W/m.k)	1:2:3 (W/m.k)	2:3:1 (W/m.k)
0% CSNTNF	0.5	0.508	0.509	0.51	0.482	0.512	0.513
0% GSNTNF	0.516	0.518	0.517	0.59	0.501	0.517	0.519
50% CSNTNF	0.501	0.512	0.513	0.514	0.487	0.517	0.516
50% GSNTNF	0.516	0.518	0.517	0.59	0.501	0.517	0.519
75% CSNTNF	0.514	0.515	0.517	0.519	0.491	0.517	0.519
75% GSNTNF	0.516	0.518	0.517	0.59	0.501	0.517	0.519

The density of CSNTNF and GSNTNF were determined using a pycnometer at a room temperature. Density of various mixture ratio of CSNTNF and GSNTNF were analysed and reported in Table 5.

**Table 5:** Density of the CSNTNF and GSNTNF for different mixture ratios of Fe:Cu:Fe_2_O_3_, and base fluid compositions at 25^o^C.

Ethylene Glycol Concentration	1:1:1 (kg/m^3^)	2:1:1 (kg/m^3^)	1:2:1 (kg/m^3^)	1:1:2 (kg/m^3^)	3:2:1 (kg/m^3^)	1:2:3 (kg/m^3^)	2:3:1 (kg/m^3^)
0% CSNTNF	1029	1038	1065	1065	1057	1067	1050
0% GSNTNF	1028	1036	1059	1063	1053	1065	1048
50% CSNTNF	1057	1035	1062	1064	1053	1065	1046
50% GSNTNF	1055	1030	1058	1061	1050	1063	1044
75% CSNTNF	1051	1031	1059	1060	1052	1059	1043
75% GSNTNF	1045	1025	1057	1059	1050	1058	1039

The viscosity of CSNTNF and GSNTNF were measured using a Brookfield DV-I PRIME digital viscometer at a temperature range of 25 °C to 55 °C. The instrument was calibrated prior to the experiment to guarantee the exactness and reliability of the measurements. The viscosity of various mixture ratios of CSNTNF and GSNTNF was reported in Table 6, and an increase in the viscosity of the NFs was observed with an increase in ethylene glycol concentration as well as mixture ratio.

**Table 6:** Viscosity of the CSNTNF and GSNTNF for different mixture ratios of Fe:Cu:Fe_2_O_3_ and base fluid compositions at 25^o^C.

Ethylene Glycol Concentration	1:1:1 (mPa.s)	2:1:1 (mPa.s)	1:2:1 (mPa.s)	1:1:2 (mPa.s)	3:2:1 (mPa.s)	1:2:3 (mPa.s)	2:3:1 (mPa.s)
0% CSNTNF	1.11	1.3	1.23	1.16	1.36	1.49	1.53
0% GSNTNF	1.07	1.21	1.17	1.14	1.26	1.36	1.49
50% CSNTNF	4.43	4.48	4.52	4.56	4.69	4.82	4.88
50% GSNTNF	4.39	4.46	5.48	4.49	4.65	4.76	4.85
75% CSNTNF	12.89	12.94	12.98	13.02	12.95	13.28	13.34
75% GSNTNF	12.84	12.89	12.92	12.97	12.89	12.19	13.28

### 4.2 Optimum mixture ratio and base fluid composition of the CSNTNF

A comparison of the performance of the AHU heater is performed for the multiple proportions of the mixture ratios of the TNF. The composition of the TNF’s base fluid is also compared to indicate the optimum TNF mixture ratio and base fluid composition in the AHU heater during winter and summer.

**Fig 9:**
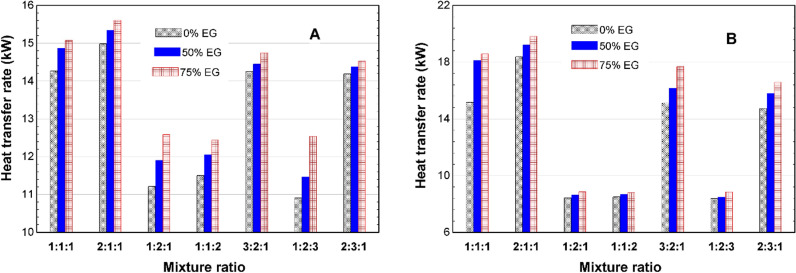
Heat transfer rate in the AHU heater in (A) winter and (B) summer.

Fig 9A illustrates that, during winter, the highest heat transfer rate is achieved with the TNF containing a 2Fe:1Cu:1Fe₂O₃ mixture ratio. The heat transfer rate reaches approximately 15 kW for the 0% EG base fluid composition and around 15.5 kW for the 75% EG composition.

Similarly, Fig 9B shows that the TNF with the 2Fe:1Cu:1Fe₂O₃ mixture ratio also exhibits the highest heat transfer rate during summer. Moreover, the 75% EG composition achieves a heat transfer rate of approximately 19.8 kW, making it the best-performing EG composition.

**Fig 10:**
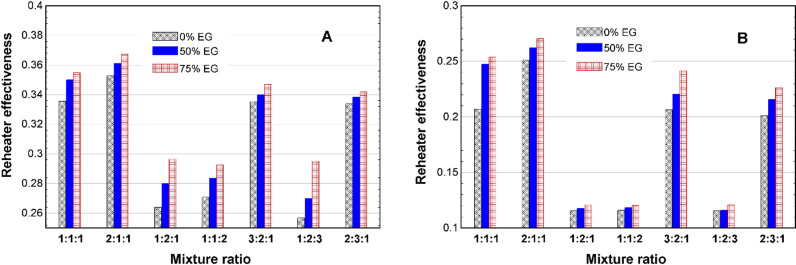
AHU heater effectiveness in (A) winter and (B) summer.

The observed seasonal variation in heater effectiveness with a 2Fe:1Cu:1Fe_2_O_3_ mixture ratio, as shown in Fig 10(A-B), can be attributed to differences in ambient conditions and system demands. In winter, the higher effectiveness (0.37) suggests improved heat transfer due to the significant temperature gradient between the heating element and the surrounding environment. The presence of a 75% EG composition likely enhances thermal stability and prevents freezing, further optimizing heat transfer efficiency.

In contrast, the reduced effectiveness in summer (0.27) may be due to the lower heating demand and more minor temperature differences, which limit the system’s ability to achieve the same level of heat exchange. Additionally, while the high EG concentration improves thermal conductivity, it also increases viscosity, potentially influencing overall flow dynamics and heat transfer rates.

**Fig 11:**
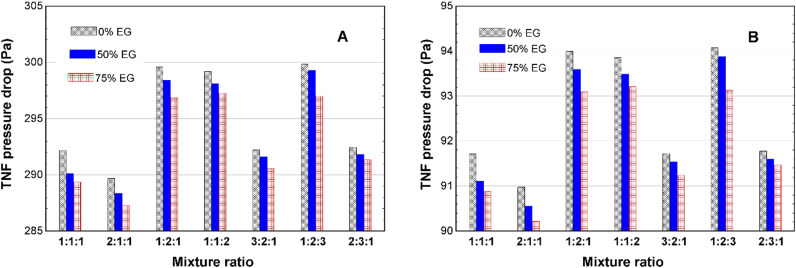
TNF pressure drop in the AHU heater in (A) winter and (B) summer.

Fig 11(A-B), shows that the TNF pressure drop will be lowest in the 2Fe:1Cu:1Fe2O3 mixture ratio with 75% EG concentration fluid. The lowest value in winter is 270 Pa; during summer, the lowest pressure drop is 90 Pa.

**Fig 12:**
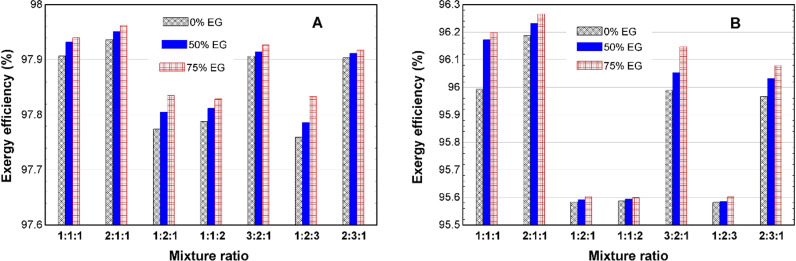
Exergy efficiency of the AHU heater in (A) winter and (B) summer.

Fig 12, shows that the exergy efficiency of the heater will be highest for the 2Fe:1Cu:1Fe_2_O_3_ mixture ratio and 75% EG composition TNF. According to Fig 12A, the highest exergy efficiency in winter is around 98%, while according to Fig 12B, the highest exergy efficiency is around 96% in summer.

### 4.3 Comparison of thermophysical properties of the CSNTNF and GSNTNF

**Fig 13:**
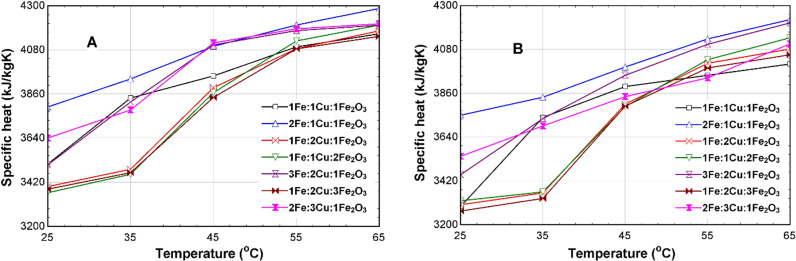
Specific heat of the 75% EG TNF at different mixture ratios for (A) synthetic and (B) green nanoparticles.

Fig 13(A-B), displays the comparison of the specific heats of the 75% EG CSNTNF and GSNTNF at different mixture ratios. The results illustrate that there is only a slight difference in the specific heats across all the mixture ratios. For the optimum 2Fe:1Cu:1Fe_2_O_3_ mixture ratio, at 35^o^C, the specific heat of the CSNTNF is 3934.4 kJ/kgK while the specific heat of the GSNTNF is 3841.1 kJ/kgK, representing a 2.8% difference.

**Fig 14:**
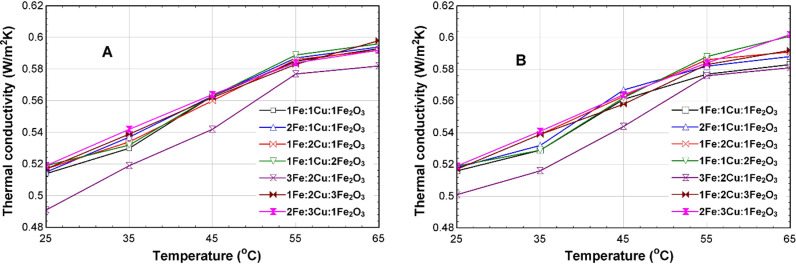
Thermal conductivity of the 75% EG TNF at different mixture ratios for (A) CSNTNF and (B) GSNTNF.

Fig 14(A-B), displays the comparison of the thermal conductivities of the 75% EG CSNTNF and GSNTNF at different mixture ratios. The results also show that, at 35^o^C, the thermal conductivity of the CSNTNF is 0.536 W/mK while the thermal conductivity of the GSNTNF is 0.532 W/mK, for the optimum 2Fe:1Cu:1Fe_2_O_3_ mixture ratio. Sun et al. (2024) reported that incorporating nanofluids (NFs) can greatly enhance the heat conduction capacity of mineral coolants, aligning with our findings.

**Fig 15:**
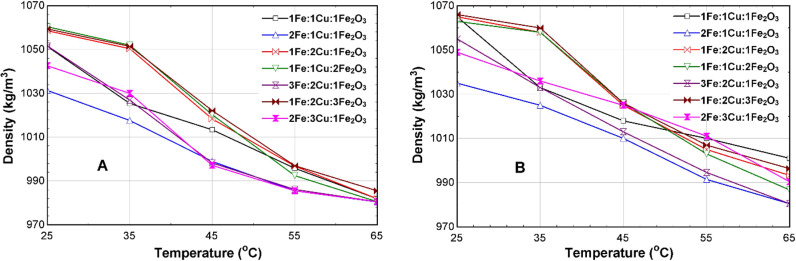
Density of the 75% EG TNF at different mixture ratios for (A) CSNTNF and (B) GSNTNF.

Fig 15(A-B), displays the comparison of the densities of the 75% EG CSNTNF and GSNTNF at different mixture ratios. The results again show a slight difference in the value of the densities. For the optimum 2Fe:1Cu:1Fe_2_O_3_ mixture ratio, at 35^o^C, the density of the CSNTNF is 1017.0 kg/m^3,^ while the density of the GSNTNF is 1024.9 kg/m^3^.

**Fig 16:**
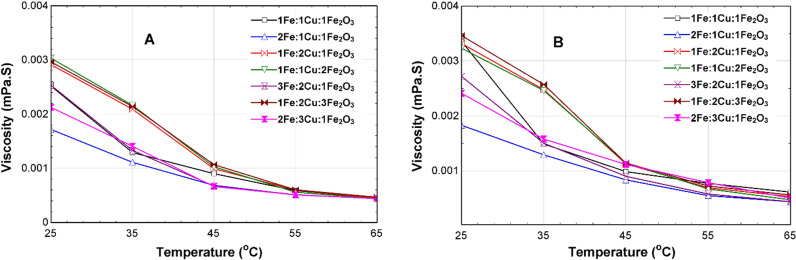
Viscosity of the 75% EG TNF at different mixture ratios for (A) CSNTNF and (B) GSNTNF.

Fig 16A, displays the comparison of the viscosities of the 75% EG CSNTNF and GSNTNF at different mixture ratios. The results also show that, at 35^o^C, the viscosity of the CSNTNF is 0.01093 mPa.S, while the viscosity of the GSNTNF is 0.001266 mPa.S, for the optimum 2Fe:1Cu:1Fe_2_O_3_ mixture ratio (Fig 16B). Adun et al. revealed changes in the dynamic viscosity of Al_2_O_3_-ZnO-Fe_3_O_4_ ternary hybrid NFs with various mixing ratios [43].

### 4.4 Comparison of the performance of the CSNTNF and GSNTNF in the AHU heater

**Fig 17:**
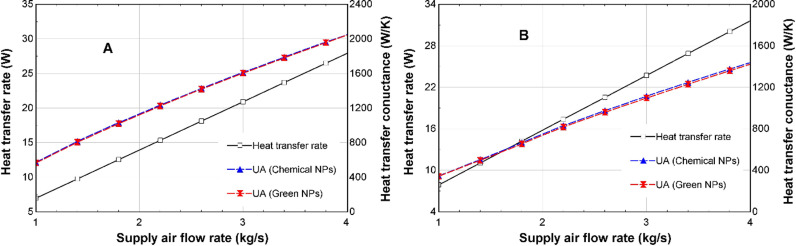
Comparison of the effect of variation of supply air flow rate on heat transfer rate and heat transfer conductance for the CSNTNF and GSNTNF in (A) winter and (B) summer.

Fig 17(A-B), shows that an increase in the supply air mass flow rate will lead to an increase in the heat transfer rate in the heater in both winter and summer. An increase from 1 kg/s to 4 kg/s causes the heat transfer rate to increase from 6.78 kW to 27.9 kW during winter and from 7.80 kW to 31.6 kW during summer. Fig 17A, also displays the effect of the variation in the supply air mass flow rate on the heat transfer conductance in winter and summer, respectively. There is an increase from 561 W/K to 2035 W/K in winter and from 344 W/K to around 1431 W/K in summer. It can also be observed from Fig 17B, that there is no difference in the heat transfer rate and heat transfer conductance for both CSNTNF and GSNTNF.

**Fig 18:**
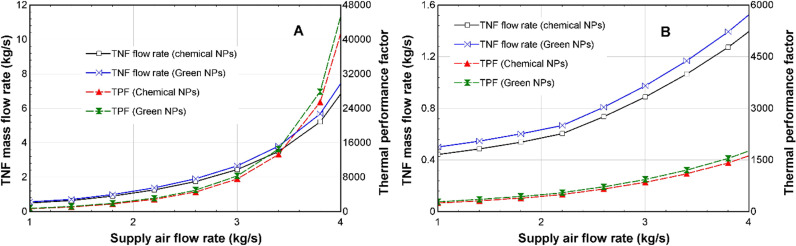
Comparison of effect of variation of supply air flow rate on TNF mass flow rate and TPF for the CSNTNF and GSNTNF in (A) winter and (B) summer.

Fig 18(A-B), shows the effect of the supply air flow rate alteration on the TNF mass flow rate in the heater. This is the amount of the TNF that will need to be supplied to the heater to keep the supply air temperatures at the design values (28^o^C in winter and 17^o^C in summer). According to Fig 18A, during winter, the CSNTNF mass flow rate will increase from 0.5 kg/s to 6.8 kg/s, while the GSNTNF mass flow rate will increase from 0.5 kg/s to 7.3 kg/s. According to Fig 18B, during summer, the CSNTNF mass flow rate will increase from 0.4 kg/s to 1.4 kg/s, while the GSNTNF mass flow rate will increase from 0.5 kg/s to 1.5 kg/s. The TPF values also shown in Fig 18A and Fig 18B will also increase as the supply air flow rate increases. The TPF for the CSNTNF increases from 811 to 41239, and the TPF of the GSNTNF increases from 811 to 45296 during winter (Fig 18A). A similar trend is observed in Fig 18B, where the TPF of the CSNTNF increases from 254 to 1623, while the TPF of the GSNTNF increases from 254 to 1724 during summer.

**Fig 19:**
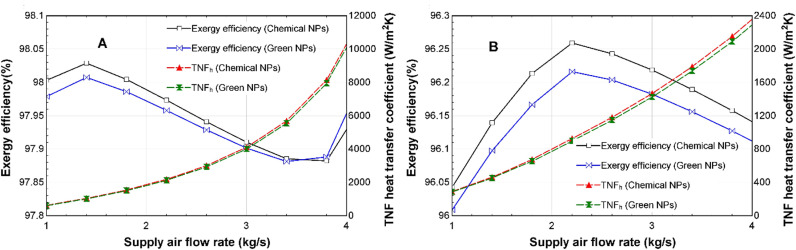
Comparison of the effect of variation of supply air flow rate on exergy efficiency and TNF heat transfer coefficient for the CSNTNF and GSNTNF in (A) winter and (B) summer.

The exergy efficiency of both the CSNTNF and GSNTNF will increase up to a maximum point but then reduce as the supplied air mass flow rate increases from 1 to 4 kg/s. This result is observed in both winter and summer, as shown in Fig 19A and Fig 19B, respectively. The exergy efficiency reaches a peak value of around 98% at a supply air flow rate of 1.4 kg/s during winter, and it reaches a peak of around 96% at a supply air flow rate of 2.2 kg/s during summer. Similarly, Chaudhari et al. highlighted that the dispersion of nano-oxides presents a promising approach to improving energy efficiency and minimizing dependence on traditional energy sources in thermal systems [44]. In addition, Fig 19A and Fig 19B, also show that the heat transfer coefficients of both the CSNTNF and GSNTNF will increase steadily as the supply air flow rate increases.

According to Fig 19A, during winter, the heat transfer coefficient increases from around 608 W/m^2^K to around 100490 W/m^2^K, while the value increases from around 290 W/m^2^K to around 2380 W/m^2^K during summer, according to Fig 19B.

## 5. Conclusion

This study investigated the performance improvement of an HVAC system utilizing water and ethylene glycol-based ternary NFs enhanced with Fe-Cu-Fe_2_O_3_ NPs. The NPs were synthesized and characterized using both chemical and green synthesis methods and incorporated into water-ethylene glycol base fluid at various mixture ratios. The experimental evaluation of thermophysical properties, coupled with numerical simulations of an air-handling unit (AHU) under Mediterranean climatic conditions, provided several important insights.

The ternary nanofluid with a 2Fe:1Cu:1Fe_2_O_3_ mixture ratio demonstrated the highest thermal performance. Compared to the base fluid, it improved heat transfer rates by up to 20% in summer and 15% in winter. This mixture ratio also achieved optimal exergy efficiency, peaking at 98% during winter operation and 96% during summer. Despite its enhanced heat transfer capabilities, the nanofluid introduced minimal increases in pressure drop and pumping power requirements, ensuring operational efficiency was maintained. The fluid exhibited excellent stability, making it suitable for long-term HVAC applications.

A comparative analysis showed that the GSNTNF performed comparably to chemically synthesized NFs, CSNTNF, with only minor differences in their thermophysical properties and performance. This positions GSNTNF as a sustainable alternative that minimizes environmental impact while delivering comparable efficiency.The study demonstrated the potential of green-synthesized ternary nanofluids (NFs) in enhancing HVAC system performance in specific locations. However, further research is needed across different locations to compare performance and to formulate other NFs using various nanoparticles, mixing ratios, and base fluids.

**Table:** 

Nomenclature	
Cp	Specific heat capacity (J/g.K)
ΔEx	Exergy gain or loss rate (W)
*F*	Friction factor
h	Convective heat transfer coefficient (W/m²·K)
*H*	Specific enthalpy (kg/s)
*K*	Thermal conductivity (W/ m·K)
$k_{f}$	Fluid’s thermal conductivity (W/m·K)
$\dot{m}$	Mass flow rate (kg/s)
Nu	Nusselt number
*P*_*P*_	Pumping power (W)
${\Delta p}_{f}$	Pressure drop (Pa)
Re	Renold number
$R_{t}$	Total thermal resistance (K/W)
${\dot{S}}_{gen}$	Entropy generation (W/K)
T	Temperature (°C)
$T_{0}$	Dead state temperature (K)
TPF	Thermal performance factor
$\dot{Q}$	Heat transfer rate (W)
U	Global heat transfer coefficient (W/m²·K)
$\eta_{II}$	Exergetic efficiency
Φ	Volume fraction (%)
Ρ	Density (g/m^3^ & kg/ m^3^)
µ	Fluid viscosity (mPa.s)
ε	Effectiveness
**Greek symbol**	
Φ	Volume fraction (%)
Ρ	Density (g/m^3^ & kg/ m^3^)
µ	Fluid viscosity (mPa.s)
**Subscripts**	
ε	Effectiveness

## Abbreviations

AHU: Air-handling unit
ALOE: Ant Lion Optimizer with Enhancements
CFD: Computational fluid dynamics
COP: Coefficient of performance
CSNTNF: Chemically synthesized ternary nanofluids
D3QN: Double Dueling Deep Q-learning
EES: Engineering equation solver
EDS: Energy-dispersive X-ray spectrometry
EG: Ethylene glycol
EG-W: Ethylene glycol-water
FTIR: Fourier Transform Infrared Spectrophotometer
GHG: Greenhouse gas
GSNTNF: Green synthesized ternary nanofluids
HVAC: Heating, ventilation, and air conditioning
MPC: Model Predictive Control
MWCNT: Multi-walled carbon nanotube
NFs: Nanofluids
NPs: Nanoparticles
RH: Relative humidity
SEM: Scanning electron microscope
TEM: Transmission electron microscope
TNF: Ternary nanofluid
W: Water
XRD: X-ray diffractometer
